# Classification of chest X-ray images by incorporation of medical domain knowledge into operation branch networks

**DOI:** 10.1186/s12880-023-01019-0

**Published:** 2023-05-09

**Authors:** Takumasa Tsuji, Yukina Hirata, Kenya Kusunose, Masataka Sata, Shinobu Kumagai, Kenshiro Shiraishi, Jun’ichi Kotoku

**Affiliations:** 1 Graduate School of Medical Care and Technology, Teikyo University, 2-11-1 Kaga, Itabashi-Ku, Tokyo, 173-8605 Japan; 2 Ultrasound Examination Center, Tokushima University Hospital, 2-50-1, Kuramoto, Tokushima, Japan; 3 Department of Cardiovascular Medicine, Tokushima University Hospital, 2-50-1, Kuramoto, Tokushima, Japan; 4 Central Radiology Division, Teikyo University Hospital, 2-11-1 Kaga, Itabashi-Ku, Tokyo, 173-8606 Japan; 5 Department of Radiology, Teikyo University School of Medicine, 2-11-1 Kaga, Itabashi-Ku, Tokyo, 173-8605 Japan

**Keywords:** Attention mechanism, Chest X-ray images, Convolutional neural networks, Deep learning, Explainable AI

## Abstract

**Background:**

This study was conducted to alleviate a common difficulty in chest X-ray image diagnosis: The attention region in a convolutional neural network (CNN) does not often match the doctor’s point of focus. The method presented herein, which guides the area of attention in CNN to a medically plausible region, can thereby improve diagnostic capabilities.

**Methods:**

The model is based on an attention branch network, which has excellent interpretability of the classification model. This model has an additional new operation branch that guides the attention region to the lung field and heart in chest X-ray images. We also used three chest X-ray image datasets (Teikyo, Tokushima, and ChestX-ray14) to evaluate the CNN attention area of interest in these fields. Additionally, after devising a quantitative method of evaluating improvement of a CNN’s region of interest, we applied it to evaluation of the proposed model.

**Results:**

Operation branch networks maintain or improve the area under the curve to a greater degree than conventional CNNs do. Furthermore, the network better emphasizes reasonable anatomical parts in chest X-ray images.

**Conclusions:**

The proposed network better emphasizes the reasonable anatomical parts in chest X-ray images. This method can enhance capabilities for image interpretation based on judgment.

## Background

In the field of analyzing clinical images such as radiological, ophthalmic, and pathological images, a great deal of interest has arisen in using convolutional neural networks (CNN) for diagnosis assistance systems used by doctors. For instance, for simple screening methods for chest disease that are reliant on chest X-ray images, some studies have found that the diagnostic accuracy achieved using CNNs is equivalent to that provided by human physicians [1]. Other studies examining the detection of recent outbreaks of the novel coronavirus disease (nCOVID-19) have been reported [2–6].

Generally, human users have difficulty interpreting CNNs, which are complex nonlinear functions. Class activation mapping (CAM) was introduced to overcome difficulties that hinder the visualization of the region of interest (ROI) used for decision-making [7]. Many alternative methods have been proposed since CAM’s introduction: Grad-CAM uses gradient information [8]; Smooth Grad produces a sensitivity map of input images with Gaussian noise and then averages them [9]; additionally, LIME [10] and SHAP [11] can approximate fundamentally important parts of images that are used when making treatment decisions.

Reportedly, CNNs do not always specifically examine appropriate regions, even when the network achieves high classification accuracy. For instance, regarding the classification of skin lesions, a case arose in which a CNN learned to judge a ruler line located near a lesion as malignant instead of the lesion site [12]. When classifying pneumonia on chest X-ray images, emphasis assigned by the CNN to metal markers at the image corners has been reported [13].

Results obtained from these earlier studies underscore that a machine's emphasis does not always match a doctor's attention region. Such findings are not surprising: earlier research efforts have not naturally incorporated domain knowledge into neural networks. Nevertheless, this important shortcoming can undermine the reliability of artificial intelligence (AI) when used for clinical applications.

Experienced medical doctors often follow specific patterns when reading medical images. For the improvement of medical image analysis, some studies of the incorporation of such medical knowledge into AI have been proposed [14].

Using a CNN, these patterns followed by experienced doctors when reading can create a model that imitates a doctor's techniques for making a diagnosis based on medical images. For example, expert doctors typically take a three-step approach when reading chest X-ray images: first viewing the entire image, concentrating on a local lesion, and finally combining the general and local information to draw inferences and make decisions [15]. One CNN approach, Dual-Ray Net, simultaneously addresses front and lateral chest X-ray images, mimicking an expert doctor's reading pattern [16]. Similarly, incorporating patterns that are typically used by expert doctors into the CNN model has improved its classification accuracy for mammography [17] and skin lesion [18] images.

Experienced medical doctors also intensively examine a few specific areas when they read medical images. Consequently, incorporating their attention regions might improve disease diagnoses that are made using medical images. This domain knowledge can be incorporated into a CNN by the application of an attention map representing the observational techniques of experienced doctors, who devote careful attention to their work. For example, introducing an attention map representing the areas which ophthalmologists specifically examine when reading fundus images has raised the respective classification accuracies for glaucoma [19] and diabetic retinopathy [20]. Other examples incorporating attention maps of medical doctors have been reported for breast cancer and melanoma screenings.

Experienced medical doctors devote attention to anatomical priors when they read medical images. This domain knowledge can be incorporated by application of an attention map to which expert doctors devote attention when reading medical images. Anatomy X-Net has achieved state-of-the-art thoracic disease classification of chest X-ray images by incorporating a lung and heart mask as an attention map into its architecture [21–25], and also by incorporating anatomical lung priors into CNN. These reports have described methods of incorporating expert doctors' pattern-reading for medical images as domain knowledge into CNN. Nevertheless, these studies did not evaluate improvement of the model's focus area to emphasize medically plausible parts.

This study proposes a method for inputting medical information into a CNN as prior information. This method forces CNNs to examine plausible areas of interest in terms of medical knowledge. Our base model is the attention branch network [26], which improves interpretability by visualizing attention (attention map) during training and by reflecting the attention region during CNN training. By guiding the attention map to make specific examinations of anatomical structures such as the lung field and heart, which are observed closely by doctors when reading images, one can construct a CNN that emphasizes appropriate regions for domain knowledge.

## Materials and methods

### Dataset

For learning and validating the proposed method, we used three chest X-ray image datasets: the Teikyo dataset, the Tokushima dataset, and the NIH14 dataset [27]. They are explained hereinafter.

The Teikyo dataset consists of 3032 frontal chest X-ray images taken at Teikyo University Hospital, including those of 2002 normal and 1030 abnormal unique patients. Abnormal cases include the upright position, along with sitting and supine positions. This dataset was approved by the institutional ethics review board (Teikyo University Review Board 17-108-6). The need for written informed consent from patients was waived because the patient data remain anonymous.

The Tokushima dataset comprises data of 1069 patients who underwent chest X-rays and right heart catheterization at Tokushima University Hospital. This dataset has a chest X-ray image and two labels for each patient. The first label identifies the presence of pulmonary hypertension according to the most recent world symposium standards: mean PAP > 20 mmHg [28–30]. The second label denotes the presence or absence of heart failure, defined as mean pulmonary artery wedge pressure higher than 18 mmHg [31–33]. The institutional review board of the Tokushima University Hospital approved the study protocol (no. 3217–3). No patient was required to give informed consent to the study because the analyses used anonymous clinical data that were obtained after each patient had given their written consent.

To resize chest X-ray images to the CNN input size while maintaining a constant aspect ratio, a padding process was applied to fill the image with zero values so that the image width and height were equal. Then the images were resized to 224 × 224 to fit the classification model input size.

The NIH14 dataset is a large chest X-ray dataset published by the National Institute of Health Clinical Center. Many reports have described studies using this dataset to develop AI models [15, 34–38]. The NIH14 dataset comprises 112,120 chest X-ray images of 30,805 unique patients. Each radiographic image is labeled with common thorax diseases of one or more of 14 types: atelectasis, cardiomegaly, consolidation, edema, effusion, emphysema, fibrosis, hernia, infiltration, mass, nodule pleural thickening, pneumonia, and pneumothorax. The images, which were saved in a portable network graphic format (1024 × 1024), were resized to 224 × 224 for input to the classification models.

### Model architecture

An attention branch network [26], because of its superior interpretability of classification models, was used as the basis for this network study. The attention branch network consists of a feature extractor, an attention branch, and a perception branch. The feature extractor is based on VGG16 [39] or ResNet50 [40]. The attention branch is used to create an attention map using CAM. The attention map generated by the attention branch is used to weigh the feature map output from the feature extractor. The perception branch outputs the feature maps, weighted by the attention map, as the final classification result for the input.

**Fig. 1:**
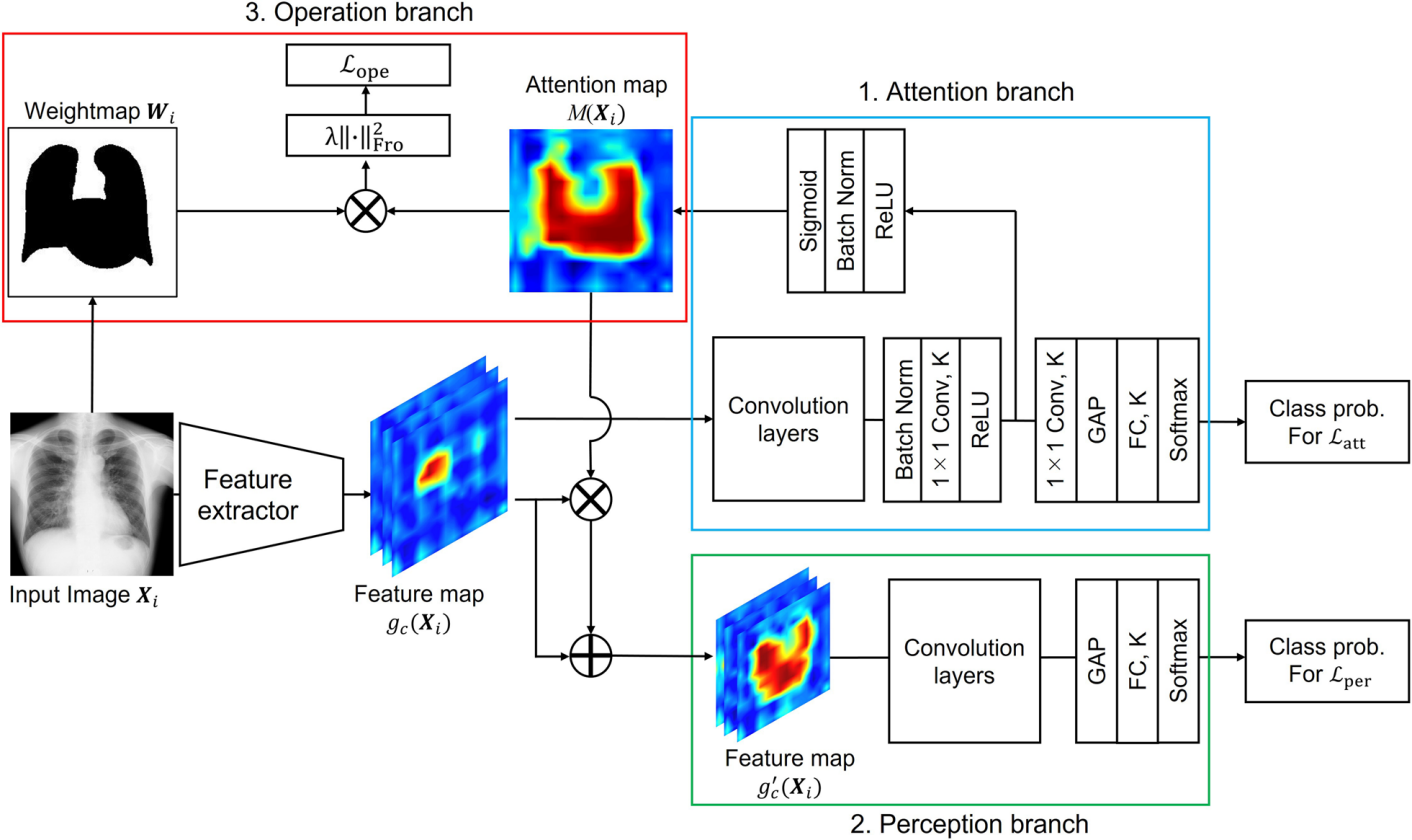
Operation branch network. The operation branch network includes feature extractors and an attention branch, perception branch, and operation branch. A chest X-ray image and a weight map showing the ROI in the image are inputs to this model

For this study, we propose a newly added operation branch, an operation branch network (OBN), to manipulate the attention map for specific examination of anatomical structures such as the lung fields and heart. This proposed network is presented in Fig. 1.

The attention branch is a structure for creating an attention map using CAM. The perception branch outputs the final probability of each class by receiving the attention and feature maps from the feature extractor. According to the following formula, the feature map is weighed by the attention map generated in the attention branch as

1 $$\begin{array}{*{20}c} {g_{c}^{^{\prime}} \left( {{\varvec{X}}_{i} } \right) = \left( {1 + M\left( {{\varvec{X}}_{i} } \right)} \right) \odot g_{c} \left( {{\varvec{X}}_{i} } \right).} \\ \end{array}$$

Here, ${{\varvec{X}}}_{i}$ represents the $i$ th input image, ${g}_{c}\left({{\varvec{X}}}_{i}\right)$ stands for the feature map from the feature extractor, $M\left({{\varvec{X}}}_{i}\right)$ denotes the attention map, ${g}_{c}^{^{\prime}}({{\varvec{X}}}_{i})$ expresses the feature map weighted by the attention mechanism, $c\in \left\{\mathrm{1,2},\cdots ,C\right\}$ is an index of the channel, and $\odot$ represents the Hadamard product [41]. The convolution layer in this Perception branch has the same structure as those of the upper layers of the ResNet50 and Densenet121 baseline models.

### Operation branch

The operation branch structure has been newly added for this study as a guide for the attention map generated from the attention branch to the correct part of the image. In the original attention branch network, the attention map generated by the attention branch is determined automatically during the learning process. Therefore, it might specifically examine regions that are inappropriate from the perspective of experts. For example, when used for chest X-ray images, the model might specifically examine regions outside the body that are not relevant at the time of diagnosis.

For this study, we introduce ${\mathcal{L}}_{\mathrm{ope}}$ as a new loss function so that the attention map will particularly examine the same anatomical structures which experienced doctors emphasize.

2 $$\begin{array}{*{20}c} {{\mathcal{L}}_{{{\text{ope}}}} \left( {{\varvec{X}}_{i} ,{\varvec{W}}_{i} } \right) = \lambda M\left( {{\varvec{X}}_{i} } \right) \odot {\varvec{W}}_{{i{\text{Fro}}}}^{2} } \\ \end{array}$$

Here, the newly added regularization term ${\mathcal{L}}_{\mathrm{ope}}\left({{\varvec{X}}}_{i},{{\varvec{W}}}_{i}\right)$: a Frobenius norm of the image (matrix) calculated using the Hadamard product of an attention map $M({{\varvec{X}}}_{i})$ and weight map ${{\varvec{W}}}_{i}$. This term imposes a penalty if the attention map emphasizes areas outside the appropriate region. Because the attention map generated from the attention branch has very fine resolution (14 × 14), we resize the image to the input size of the classification model. Then we calculate the Hadamard product.

**Fig. 2:**
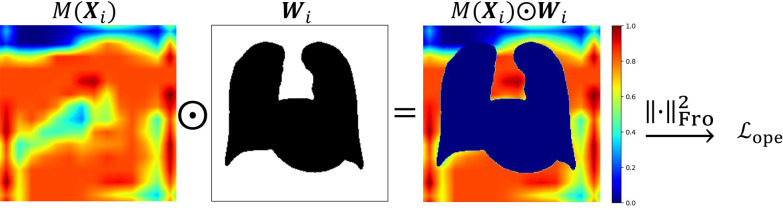
Visualization of the calculation process in the operation branch. An attention map is generated from the attention branch, a weight map, and the Hadamard product of the attention map and the weight map. White and black areas on the weight map respectively represent one and zero values. The red zone shows the highest values in an attention map

This study's weight maps are the convex hull created by lung field segmentation, lung field and heart segmentation images, and images created manually by experts. A conceptual visualization of calculation of the Frobenius norm of an attention map and a weight map is presented in Fig. 2. Regularization parameter $\lambda$ is a hyperparameter. It was tuned using grid search, which was set as {0.1, 0.01, 10^–3^}.

### Operation branch network's loss function

The loss function of the operation branch network proposed for this analysis consists of the sum of losses of attention, perception, and operation branches. The following equation is the overall loss function.

3 $$\begin{array}{c}L\left({{\varvec{X}}}_{i}, {{\varvec{W}}}_{i}\right)={\mathcal{L}}_{\mathrm{att}}\left({{\varvec{X}}}_{i}\right)+{\mathcal{L}}_{\mathrm{per}}\left({{\varvec{X}}}_{i}\right)+{\mathcal{L}}_{\mathrm{ope}}\left({{\varvec{X}}}_{i},{{\varvec{W}}}_{i}\right)\end{array}$$

In that equation, ${\mathcal{L}}_{\mathrm{att}}\left({{\varvec{X}}}_{i}\right)$ and ${\mathcal{L}}_{\mathrm{per}}\left({{\varvec{X}}}_{i}\right)$ respectively represent the loss of the attention branch and perception branch. In addition, ${{\varvec{X}}}_{i}$ denotes the $i$ th input image.

### Weight map creation

Doctors specifically examine the lung field, heart, and mediastinum during diagnostic examinations. To incorporate the anatomical information of chest X-ray images into a network, we created weight maps for these areas. The weight map has a pairing structure with the image input to the proposed model. This weight map is a binary image in which the pixel values represent the regions the proposed model wants to specifically examine and those it does not want to emphasize.

**Fig. 3:**
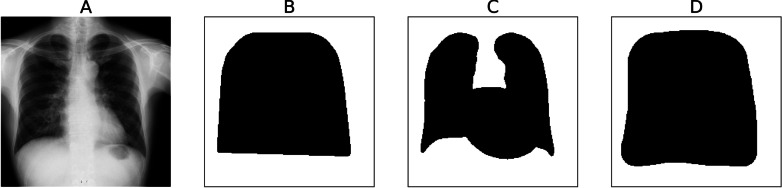
Examples of weight maps. **A**, Input image. **B**, Weight map with the convex hull on the mask lung field. **C**, Weight map combining a mask image of the lung field and heart. **D**, Weight map produced with the doctor’s support, manually masked to include the heart

For this study, we used the Unet segmentation model [42] to create the convex hull image of the lung field and the combined images of the lung field and heart. Under the direction of an experienced doctor, we manually created weight maps for the Tokushima and the Teikyo datasets to include the heart. Figure 3 presents an example of these weight maps. The weight map's black (anatomical) and white (non-anatomical) areas respectively represent zero and one values.

### Unet

We used Unet [42] to segment the lung and heart in chest X-ray images. Additionally, we used 704 chest X-ray images from the Montgomery County Chest X-ray database [43, 44] as ground truth for lung field segmentation, and 247 chest X-ray images from JSRT [45, 46] as those for the heart. Several lung segmentation studies using these databases have been reported [47–49]. These images were resized to 224 × 224 to input the classification network. Adam (alpha = 1.0 × 10^–3^, beta1 = 0.9, beta2 = 0.999) was used for training Unet with a batch size of 16. The number of epochs was set as 100. Combo Loss [50], a combination of Binary Cross-Entropy Loss and Dice Loss, was adapted for use in the segmentation task.

The Dice coefficient [51]

4 $$\begin{array}{c}\frac{2\left|X\cap Y\right|}{\left|X\right|+\left|Y\right|}\#\end{array}$$

and intersection over union (IoU) [52]

5 $$\begin{array}{c}\frac{\left|X\cap Y\right|}{\left|X\cup Y\right|}\#\end{array}$$

were used as evaluation indices for segmentation. Here, $X$ represents the region predicted by the segmentation model; $Y$ shows the region of ground truth.

This study created mask images of the lung field and heart for the Teikyo, Tokushima, and NIH 14 datasets. For lung field and heart segmentation, we performed ten-fold cross-validation. We also fine-tuned heart segmentation with a pre-trained model of lung segmentation. Then, we calculated the average output of the ten trained model's binarized output and created lung field and heart mask images for the Teikyo, Tokushima, and NIH 14 datasets. A weight map's anatomical and non-anatomical areas are respectively represented as zero and one values.

### Learning

**Fig. 4:**
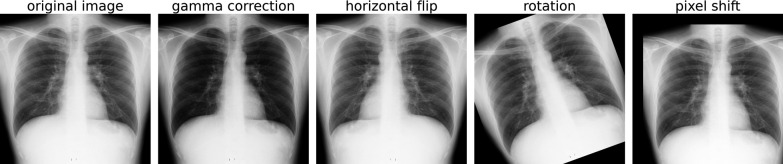
Examples of augmented images. Left, original image. Middle left, gama correction. Middle, horizontal flip. Middle right, rotation. Right, pixel shift

For this study, we built three operation branch networks based on models: Resnet50 [40] and Densenet121 [53], which were pre-trained on ImageNet [54]. Fine-tuning was performed with those models. Adam [55] used the optimization algorithm. First, 100 epoch learning was performed, with early stopping occurring to prevent overfitting when the classification accuracy for a validation dataset was the highest. We also used grid search to seek the optimal parameters for the initial value of the learning rate. This search space was set as {10^–5^, 10^–4^,10^–3^}. To reduce the influence of the imbalanced data, the inverse ratios of the number of data were weighted respectively to the cross-entropy loss of the attention branch and the perception branch. In addition, a multi-label binary cross-entropy loss was used to train the NIH14 dataset. Furthermore, all images were augmented using gamma correction, horizontal flipping, rotation, and pixel shift. Images enhanced using these techniques are presented in Fig. 4.

We built the proposed network on Reedbush-L running on a computer (Xeon CPUs; Intel Corp. and Tesla P100 16 GB GPU; NVIDIA Corp.) with a Pytorch (ver. 1.5.0) deep learning framework.

### Attention index

The final output of the attention branch network is the output obtained by inputting the attention map weighted to the feature map to the perception branch. We verified the effects of the operation branch on the Grad-CAM images. For this study, we defined a new index to evaluate how an activation site of Grad-CAM specifically examines an appropriate part in the image.

We express the degree of attention on the pixel $(i, j)$ as${p}_{i,j}$, the index set of the entire image as$\Omega$, and the index set of the ROI as$\mathrm{A}$. The total attention $I\left( {\Omega } \right)$ of the entire image can therefore be defined as shown below.

6 $$\begin{array}{c}I\left(\Omega \right)=\sum_{\left(i, j\right)\in\Omega }{p}_{i,j}\#\end{array}$$

The total attention of the trained model $\mathrm{I}\left(\mathrm{A}\right)$ is defined as

7 $$\begin{array}{c}I\left(\mathrm{A}\right)=\sum_{\left(i, j\right)\in \mathrm{A}}{p}_{i,j}.\#\end{array}$$

Therefore, we can define the Attention Index ${\mathrm{I}}_{\mathrm{A}}$ as

8 $$\begin{array}{c}{I}_{\mathrm{A}}=\frac{I(\mathrm{A})}{I(\Omega )}.\#\end{array}$$

This study uses this index to test our algorithm’s performance.

## Results

### Unet

First, we explain the results of segmentation learning of the lung field and heart using Unet to create weight maps showing the ROI in the chest X-ray image. Ten-fold cross-validation was applied for segmentation of the lung field and heart. Table 1 presents the mean values and standard deviations of accuracy, IoU, and the Dice coefficient found from ten-fold cross-validation.

**Table 1:** Ten-fold cross-validation results of Unet

	Accuracy [%]	IoU	Dice coefficient
Lung	96.78 ± 2.52	0.92 ± 0.05	0.96 ± 0.0.3
Heart	96.44 ± 3.32	0.79 ± 0.09	0.88 ± 0.06

### Ten-fold cross-validation

For this study, we used three chest X-ray datasets to investigate the operation branch effects: the Teikyo University dataset, the University of Tokushima dataset, and the NIH14 dataset. They are used to guide the focus of attention.

**Fig. 5:**
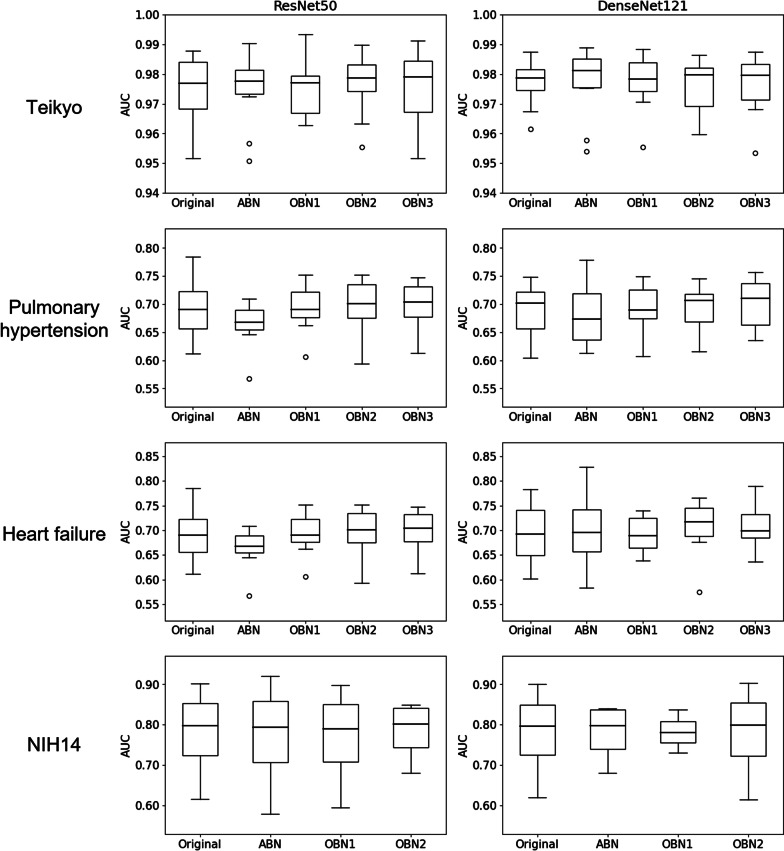
Box plots of learning results. Left column, ResNet50. Right column, DenseNet121. Original, original CNN. ABN, attention branch network. OBN1, Operation branch network using the weight map with a convex hull on mask images of the lung field. OBN2, Operation branch network using weight maps with combined mask images of the lung field and heart. OBN3, Operation branch network using weight maps masked manually to include the heart, produced using a doctor’s support

We evaluated learning models using ten-fold cross-validation for the Teikyo and the University of Tokushima datasets and using the hold-out method for the NIH14 dataset. Figure 5 presents classification results obtained for the Teikyo dataset and pulmonary hypertension and heart failure dataset at the University of Tokushima. The bottom figures portray boxplots of the 14 disease classification results of the NIH14 dataset using the hold-out method. These numerical classification results are presented in Tables 2, 3 and 4. A comparison of the proposed method and a state-of-the-art method with the NIH 14 dataset is presented in Table 5.

**Table 2:** Teikyo dataset classification results from ten-fold cross-validation

Backbone	Model	Accuracy [%]	Sensitivity [%]	Specificity [%]	AUC
ResNet50	Conventional	93.16 ± 1.49	89.51 ± 3.03	96.80 ± 1.19	0.97 ± 0.01
	ABN	93.54 ± 1.55	91.17 ± 2.86	95.90 ± 1.43	0.97 ± 0.01
	OBN1	93.62 ± 1.48	90.29 ± 3.19	96.95 ± 1.91	0.98 ± 0.01
	OBN2	93.44 ± 1.81	90.78 ± 2.89	96.11 ± 2.24	0.98 ± 0.01
	OBN3	93.23 ± 1.53	89.90 ± 3.23	96.55 ± 1.78	0.98 ± 0.01
DenseNet121	Conventional	93.99 ± 1.22	91.07 ± 2.29	96.90 ± 0.89	0.98 ± 0.01
	ABN	93.59 ± 1.45	90.68 ± 2.05	96.50 ± 2.07	0.98 ± 0.01
	OBN1	93.50 ± 1.51	90.19 ± 2.80	96.80 ± 1.20	0.98 ± 0.01
	OBN2	93.64 ± 1.26	90.78 ± 3.08	96.50 ± 1.21	0.98 ± 0.01
	OBN3	93.62 ± 2.03	90.19 ± 3.70	97.05 ± 1.38	0.98 ± 0.01

ResNet50 and DenseNet121 are used as backbone approaches: ABN, attention branch network; OBN1, operation branch network using weight map with a convex hull on mask images of lung field; OBN2, operation branch network using weight maps with a combined mask image of lung field and heart; OBN3, operation branch network using weight maps manually masked to include the heart, produced with a doctor's support

**Table 3:** Pulmonary hypertension classification results from ten-fold cross-validation

Backbone	Model	Accuracy [%]	Sensitivity [%]	Specificity [%]	AUC
ResNet50	Conventional	64.17 ± 5.89	68.56 ± 10.74	59.78 ± 11.48	0.69 ± 0.05
	ABN	63.10 ± 3.65	61.03 ± 7.99	65.17 ± 7.83	0.69 ± 0.04
	OBN1	63.11 ± 4.37	60.52 ± 7.86	65.70 ± 12.91	0.69 ± 0.04
	OBN2	**64.95 ± 4.66**	64.15 ± 9.21	65.74 ± 15.01	**0.70 ± 0.04**
	OBN3	64.53 ± 4.03	61.75 ± 9.53	67.31 ± 12.84	**0.70 ± 0.04**
DenseNet121	Conventional	63.66 ± 4.99	65.60 ± 6.61	61.73 ± 10.30	0.69 ± 0.05
	ABN	61.54 ± 4.94	57.72 ± 9.88	65.35 ± 11.12	0.68 ± 0.06
	OBN1	64.33 ± 4.55	61.77 ± 8.69	66.88 ± 8.50	0.69 ± 0.04
	OBN2	64.05 ± 4.11	57.40 ± 11.06	70.69 ± 5.08	0.69 ± 0.04
	OBN3	**64.79 ± 3.59**	58.70 ± 7.73	70.88 ± 6.52	**0.70 ± 0.04**

Bolded numbers indicate the highest score

ResNet50 and DenseNet121 are used as backbone approaches: ABN, attention branch network; OBN1, operation branch network using weight map with a convex hull on mask images of lung field; OBN2, operation branch network using weight maps with a combined mask image of lung field and heart; OBN3, operation branch network using weight maps manually masked to include the heart, produced with a doctor's support

**Table 4:** Heart failure classification results from ten-fold cross-validation

Backbone	Model	Accuracy [%]	Sensitivity [%]	Specificity [%]	AUC
ResNet50	Conventional	60.26 ± 3.64	37.59 ± 15.63	82.92 ± 10.41	0.68 ± 0.03
	ABN	59.20 ± 5.81	81.09 ± 17.98	67.31 ± 10.46	0.67 ± 0.06
	OBN1	62.49 ± 3.90	58.75 ± 16.44	66.24 ± 13.27	**0.70 ± 0.04**
	OBN2	**64.38 ± 2.79**	58.32 ± 8.62	70.44 ± 7.31	0.69 ± 0.03
	OBN3	61.99 ± 3.66	54.87 ± 11.32	69.11 ± 6.24	0.68 ± 0.04
DenseNet121	Conventional	61.29 ± 5.45	39.46 ± 16.01	83.12 ± 8.54	0.70 ± 0.06
	ABN	65.24 ± 5.09	61.00 ± 15.36	69.49 ± 11.55	0.70 ± 0.05
	OBN1	63.79 ± 4.30	55.65 ± 17.19	71.93 ± 11.20	0.69 ± 0.03
	OBN2	**66.54 ± 5.21**	64.40 ± 10.63	68.67 ± 5.07	**0.71 ± 0.05**
	OBN3	63.80 ± 3.47	58.03 ± 16.14	69.57 ± 15.25	0.70 ± 0.05

Bolded numbers indicate the highest score

ResNet50 and DenseNet121 are used as backbone approaches: ABN, attention branch network; OBN1, operation branch network using weight map with a convex hull on mask images of lung field; OBN2, operation branch network using weight maps with combined mask images of the lung field and heart; OBN3, operation branch network using weight maps manually masked to include the heart according to the doctor's support

**Table 5:** Results obtained using earlier methods with the NIH 14 dataset

Method	Atel	Card	Effu	Infi	Mass	Nodu	Pne1	Pne2	Cons	Edem	Emph	Fibr	PT	Hern	Mean	
Wang et al. [22]	0.716	0.807	0.784	0.609	0.706	0.671	0.633	0.806	0.708	0.835	0.815	0.769	0.708	0.767	0.738	
CheXNet [1]	0.809	0.925	0.864	0.735	0.868	0.780	0.768	0.889	0.790	0.888	0.937	0.805	0.806	0.916	0.841	
AG-CNN [10]	0.853	0.939	0.903	0.754	0.902	0.828	0.774	0.921	0.842	0.924	0.932	0.864	0.837	0.921	0.871	
Triple Attention [34]	0.779	0.895	0.836	0.710	0.834	0.777	0.737	0.878	0.759	0.855	0.933	0.838	0.791	0.938	0.826	
Anatomy X-Net [16]	0.815	0.908	0.880	0.705	0.855	0.793	0.775	0.874	0.810	0.896	0.923	0.832	0.786	0.962	0.844	
ResNet50 (Backbone)	Conventional	0.746	0.874	0.809	0.694	0.762	0.709	0.686	0.835	0.733	0.837	0.869	0.793	0.745	0.913	0.786
	ABN	0.746	0.874	0.813	0.686	0.777	0.713	0.679	0.838	0.731	0.838	0.873	0.801	0.752	0.914	0.788
	OBN1	0.745	0.884	0.812	0.691	0.773	0.708	0.674	0.837	0.729	0.834	0.867	0.811	0.751	0.908	0.787
	OBN2	0.747	0.881	0.805	0.695	0.775	0.705	0.689	0.841	0.723	0.839	0.873	0.796	0.750	0.884	0.786
DenseNet121 (Backbone)	Conventional	0.744	0.879	0.814	0.683	0.791	0.701	0.694	0.822	0.731	0.833	0.826	0.793	0.750	0.883	0.782
	ABN	0.745	0.885	0.814	0.698	0.783	0.718	0.692	0.842	0.735	0.836	0.884	0.813	0.759	0.892	0.792
	OBN1	0.743	0.885	0.811	0.692	0.764	0.710	0.684	0.848	0.728	0.837	0.881	0.808	0.756	0.905	0.789
	OBN2	0.742	0.883	0.810	0.697	0.767	0.716	0.682	0.851	0.733	0.838	0.877	0.795	0.753	0.917	0.790

The AUC of each class and the average AUC of 14 diseases are shown. These diseases for the NIH 14 dataset are atelectasis (Atel), cardiomegaly (Card), effusion (Effu), infiltration (Infi), mass (Mass), nodule (Nodu), pneumonia (Pne1), pneumothorax (Pne2), consolidation (Cons), edema (Edem), emphysema (Emph), fibrosis (Fibr), pleural thickening (P.T.), hernia (Hern), with ResNet50 and DenseNet121 used as backbone approaches. ABN, attention branch network; OBN1, operation branch network using a weight map with a convex hull on mask images of the lung field; OBN2, operation branch network using weight maps with combined mask images of the lung field and heart

The AUC of the Teikyo dataset and the NIH14 dataset classification show almost identical values for Resnet50 and Densenet121. Introducing the operation branch seemed to raise the AUC for two pulmonary hypertension and heart failure classification models in the Tokushima dataset.

### Visualization of attention maps

**Fig. 6:**
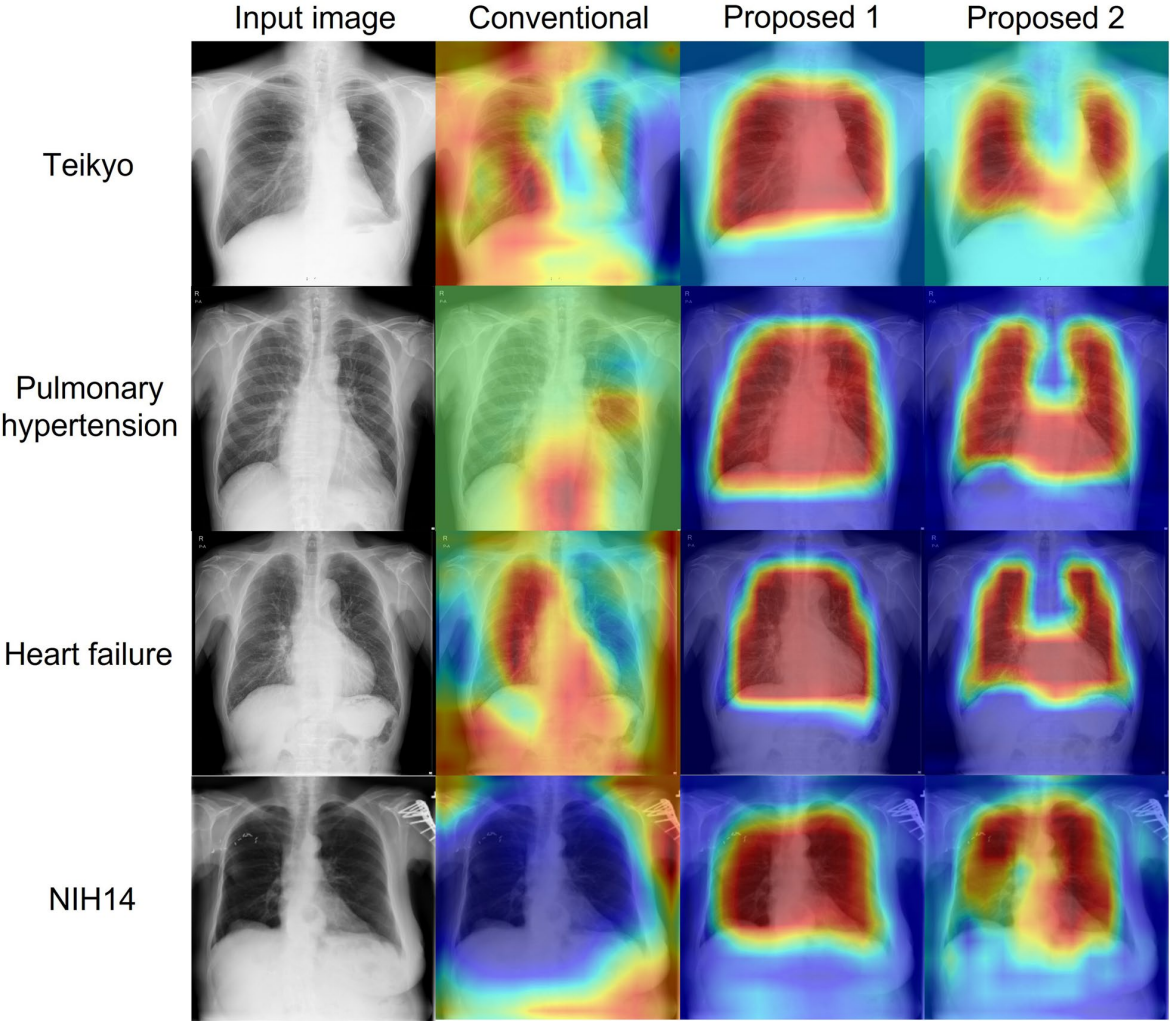
Comparison of attention maps. The upper row shows the Teikyo dataset. The upper middle row shows the Tokushima dataset pulmonary hypertension classification. The lower middle row shows the Tokushima dataset heart failure classification. The lower row presents the NIH14 dataset. Columns show the following: Far-left column, input images; left middle column, conventional attention branch network; right middle column, operation branch network using weight maps with a convex hull mask of lung field; far left column, operation branch network created using weight maps with a combined mask image of the lung field and heart

To assess the improvement of attention attributable to introduction of the operation branch in the proposed method, we compared attention maps generated by the attention branches. The attention maps of the attention branch and operation branch networks based on Densenet121 are presented in Fig. 6 for each dataset. The activation maps of the models are presented in the figure: conventional attention branch network, operation branch network using weight maps with a convex hull mask of the lung field, and operation branch network using weight maps with a lung field and heart mask.

### Evaluation of focus areas in Grad-CAM images

**Fig. 7:**
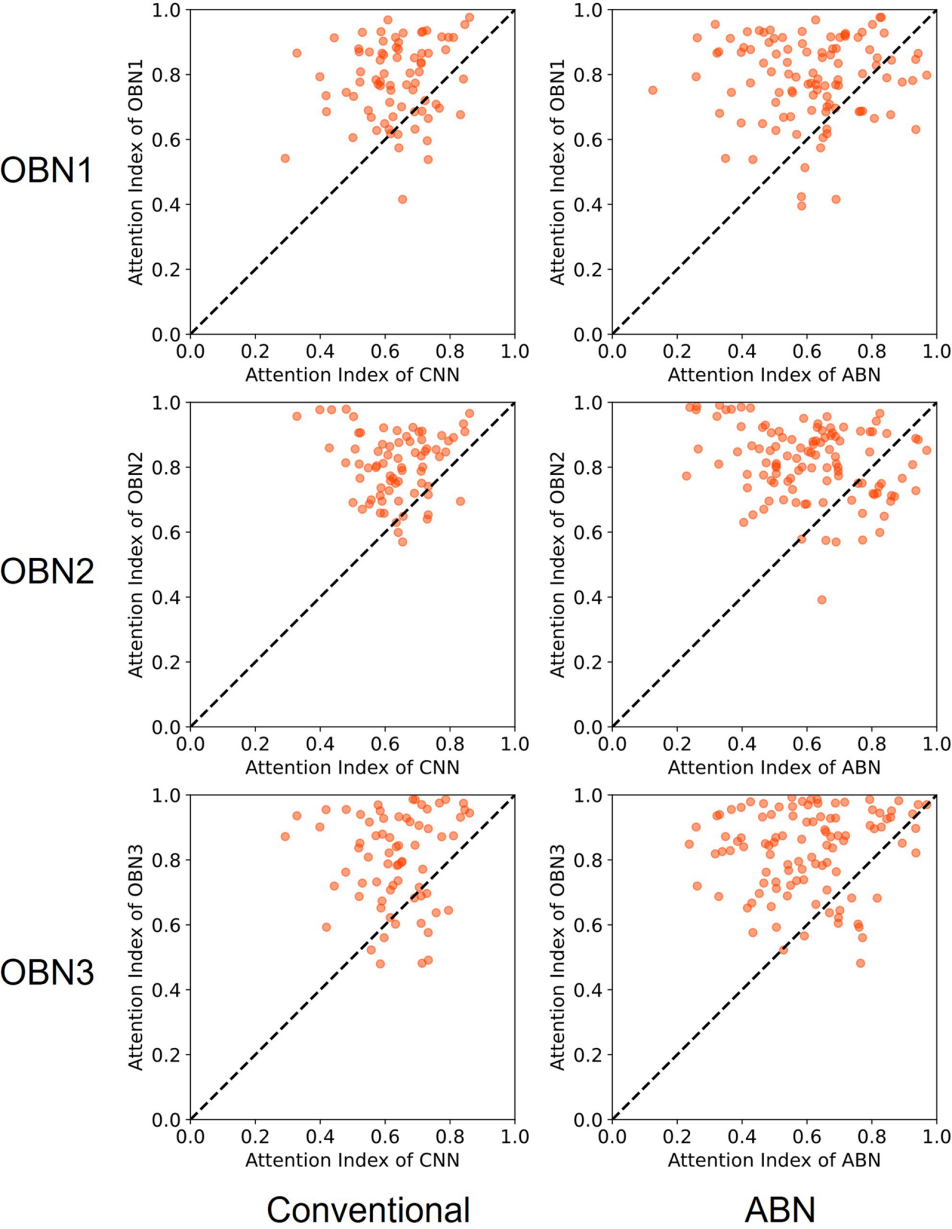
Scatter plot of Attention Index for heart failure classification in the Tokushima dataset. Left column, conventional DenseNet121. Right column, original attention branch network. The horizontal axis shows the attention index value in the attention branch network. The vertical axis shows those of others: OBN1, operation branch network using weight maps with a convex hull mask of lung field; OBN2, operation branch network using weight maps with lung field and heart mask; OBN3, operation branch network created manually by an experienced doctor using weight maps

**Fig. 8:**
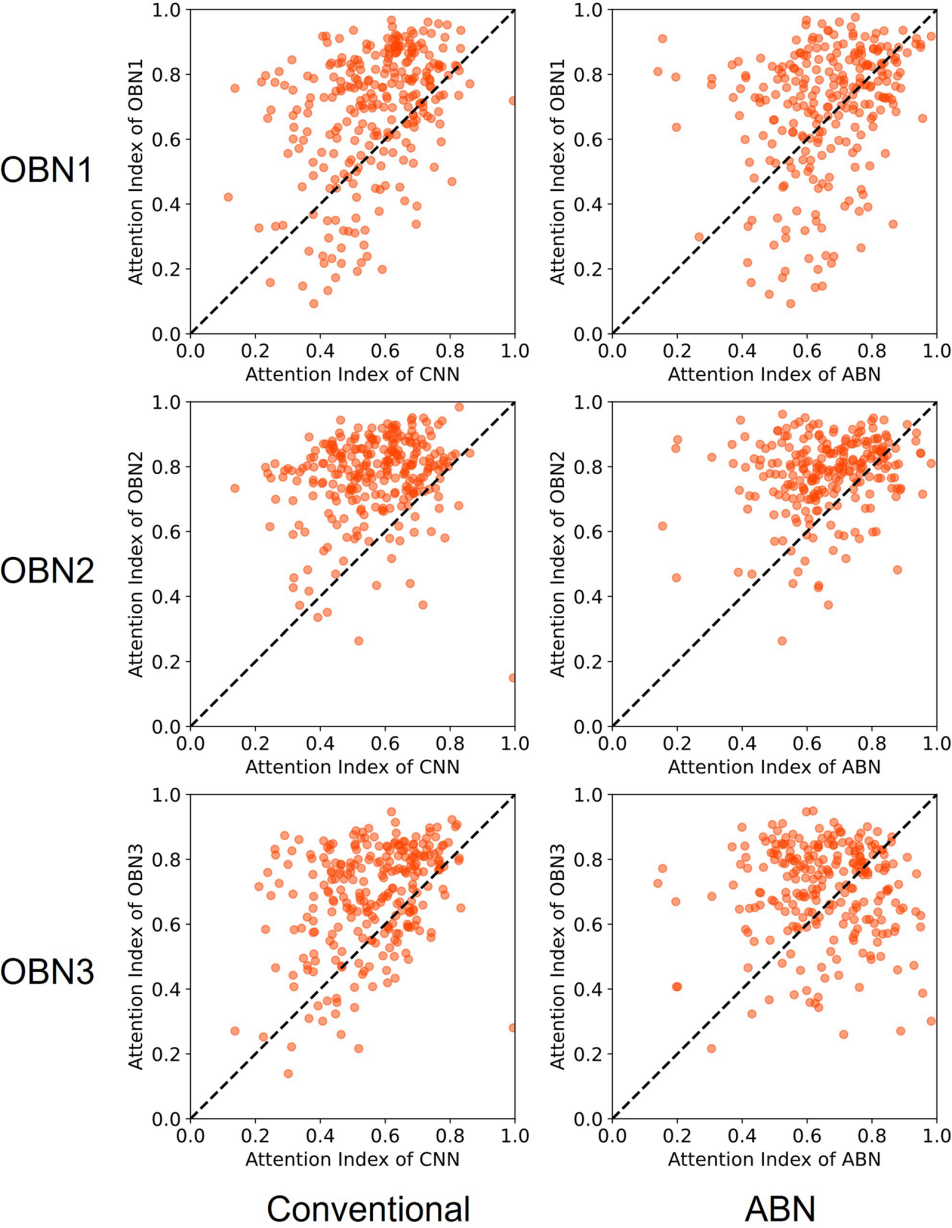
Scatter plot of attention index for pulmonary hypertension in the Tokushima dataset. Left column, conventional DenseNet121. Right column, original attention branch network. The horizontal axis shows the attention index value in the attention branch network. The vertical axis shows those of others: OBN1, operation branch network using weight maps with a convex hull mask of lung field; OBN2, operation branch network using weight maps with a combined mask of the lung field and heart; OBN3, operation branch network created manually by an experienced doctor using weight maps

**Fig. 9:**
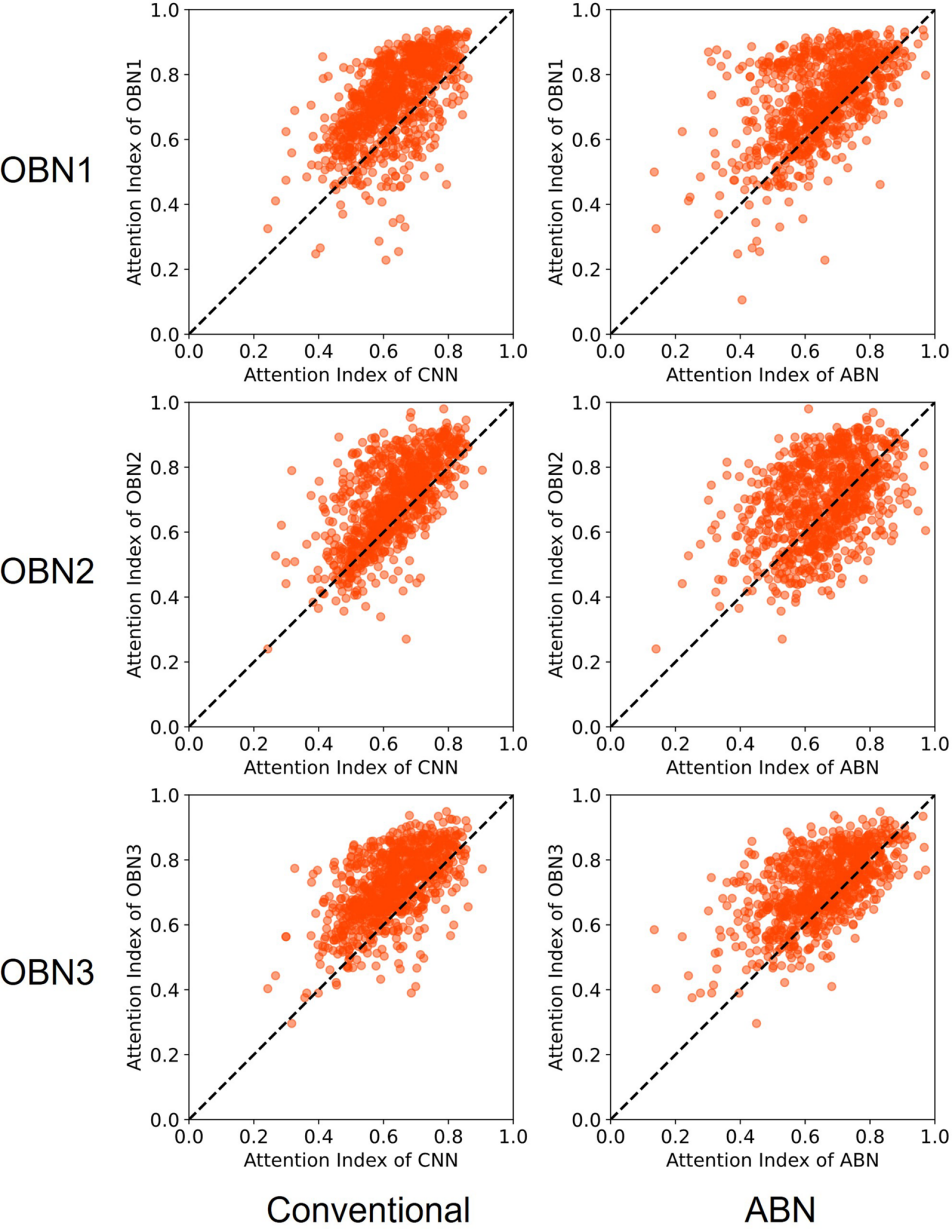
Scatter plot of attention index in Teikyo dataset. Left column, conventional DenseNet121; right column, original attention branch network. The horizontal axis shows the attention index value in the attention branch network. The vertical axis shows those of others: OBN1, operation branch network using weight maps with a convex hull mask of the lung field; OBN2, operation branch network using weight maps with a combined mask of the lung field and heart; OBN3, operation branch network created manually by an experienced doctor using weight maps

To verify effects of the operation branch on the Grad-CAM images, we calculated the attention index for Grad-CAM images based on DenseNet121 in Tokushima datasets (heart failure and hypertension) and Teikyo datasets. We present data classified as true positive in Figs. 7, 8 and 9. In these figures, the horizontal and vertical axes respectively show values of the attention index in the operation branch network and in the other models. Dots to the upper left of the diagonal show that the operation branch raised the attention index value for the conventional CNN and the original attention branch network. These numerical results for the Attention index of True positive data are presented in Table 6.

**Table 6:** Results of attention index for true positive data

Dataset	Backbone	Conventional	ABN	OBN1	OBN2	OBN3
Teikyo	ResNet50	0.60 ± 0.11	0.59 ± 0.11	0.56 ± 0.10	0.57 ± 0.11	0.58 ± 0.10
	DenseNet121	0.63 ± 0.11	0.65 ± 0.13	0.72 ± 0.13	0.69 ± 0.13	0.72 ± 0.11
Pulmonary hypertension	ResNet50	0.60 ± 0.25	0.57 ± 0.27	0.69 ± 0.23	0.70 ± 0.24	0.62 ± 0.28
	DenseNet121	0.55 ± 0.15	0.65 ± 0.16	0.68 ± 0.20	0.77 ± 0.13	0.69 ± 0.16
Heart failure	ResNet50	0.74 ± 0.25	0.73 ± 0.24	0.69 ± 0.25	0.78 ± 0.20	0.66 ± 0.23
	DenseNet121	0.75 ± 0.21	0.56 ± 0.31	0.84 ± 0.19	0.84 ± 0.21	0.90 ± 0.09

This table presents the averaged Attention index over all ten splits, with the respective calculated standard deviations for true positive data: ABN, attention branch network; OBN1, operation branch network using weight map with a convex hull on mask images of lung field; OBN2, operation branch network using weight maps with combined mask images of the lung field and heart

**Fig. 10:**
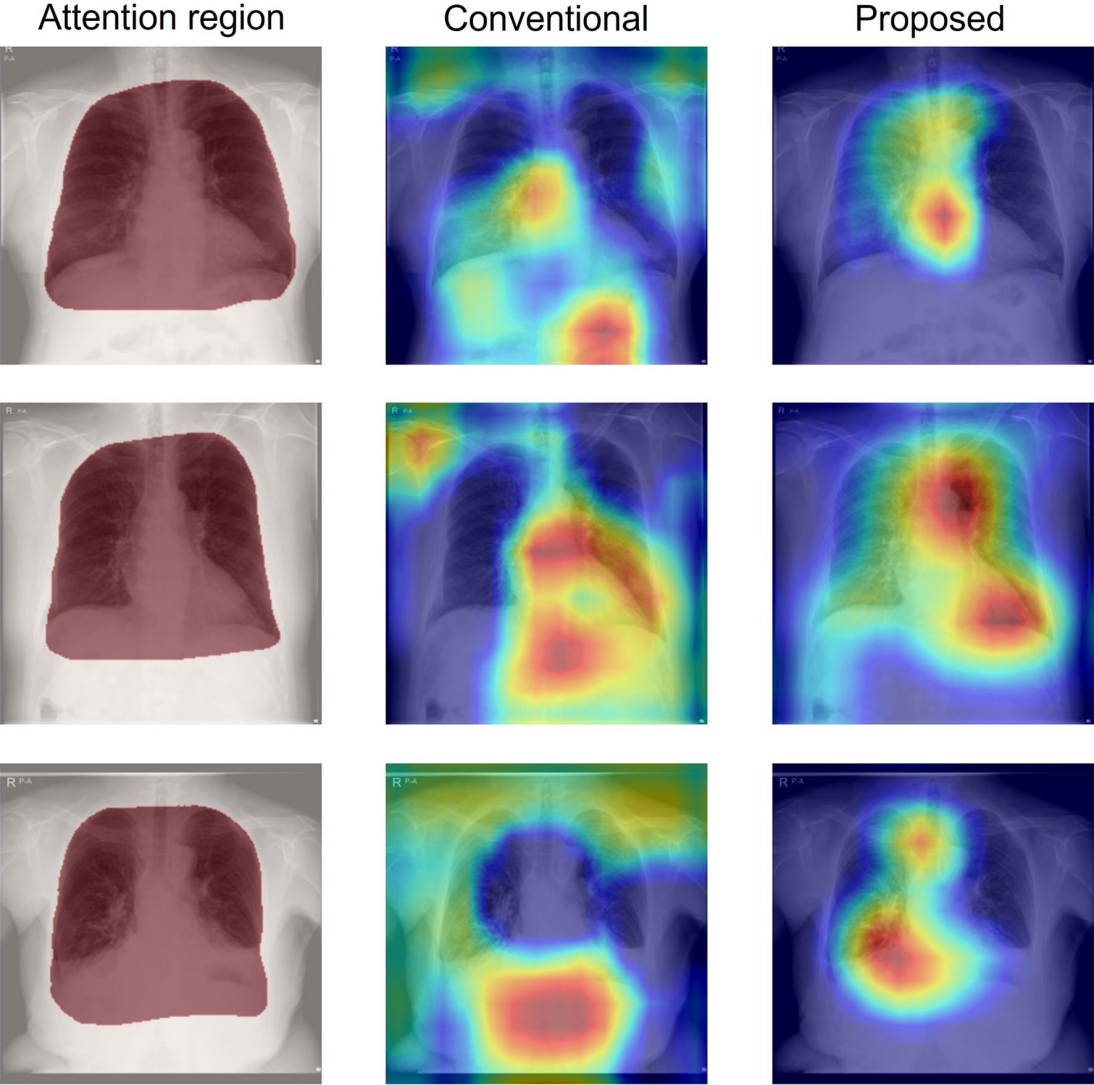
Grad-CAM images for which the attention index was raised by an operation branch. The left column (Attention region) presents input images, where red zones show the ROI. The center column (Conventional) shows activation maps of the original attention branch networks based on DenseNet121. The right column (Proposed) presents activation maps of operation branch networks based on DenseNet121 created manually by an experienced doctor using weight maps

**Fig. 11:**
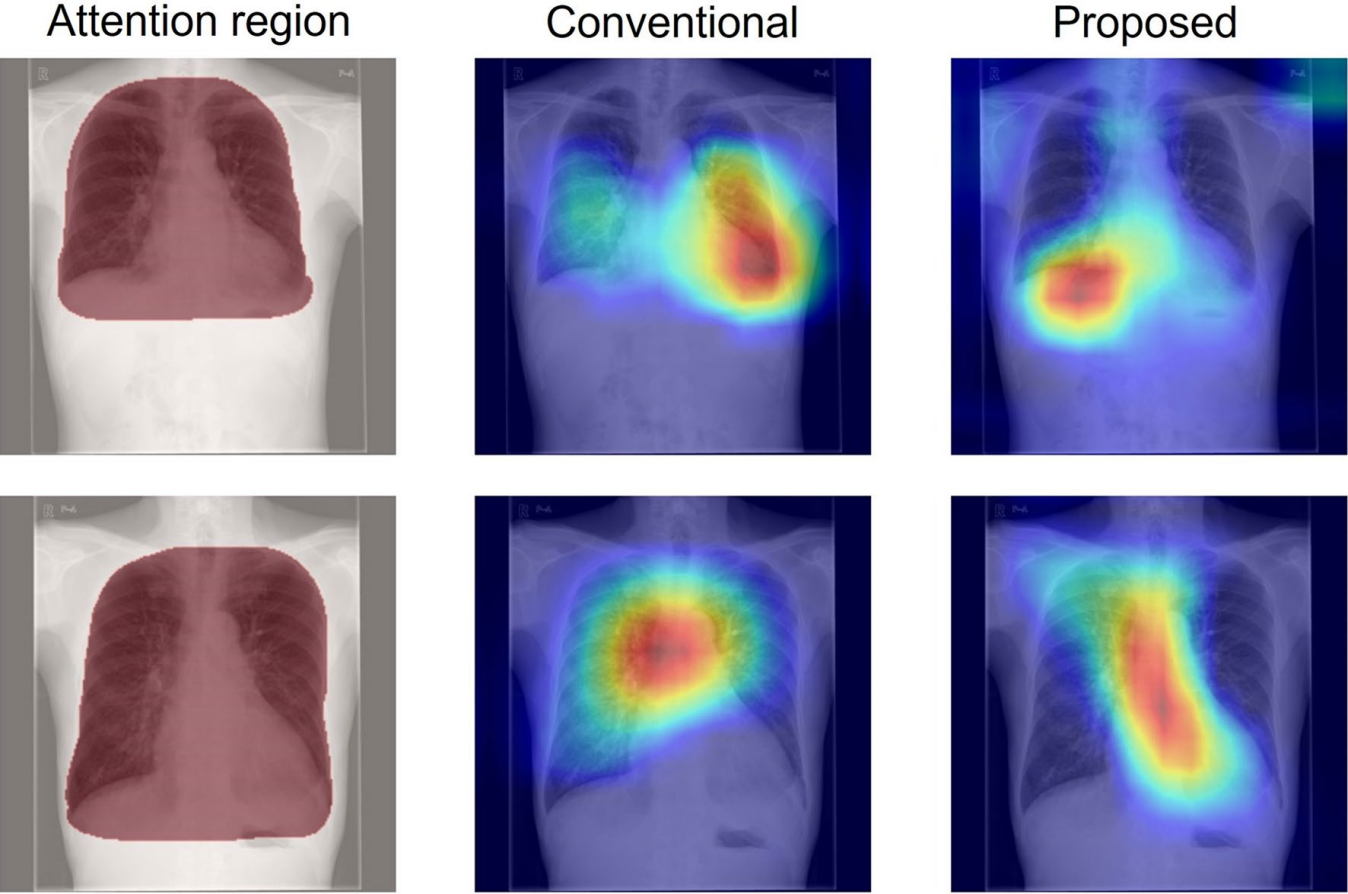
Grad-CAM images for which the attention index is decreased in the case of an operation branch. The left column (Attention region) presents input images, where red zones show the ROI. The center column (Conventional) shows activation maps of the original attention branch networks based on DenseNet121. The right column (Proposed) depicts activation maps of operation branch networks based on DenseNet121 created manually by an experienced doctor using weight maps

Figures 10 and 11 respectively present comparisons of Grad-CAM images for which the attention indexes were raised and reduced by introducing an operation branch. The left column (Attention region) shows input images superimposed on the attention region (red convex). The center column (Conventional) shows activation maps of the original attention branch networks based on DenseNet121. The right column (Proposed) presents activation maps of the operation branch network based on DenseNet121 using weight maps that were created manually through collaboration with an experienced doctor. The attention index of the operation branch network was higher than that of the attention branch network for heart failure classification using the University of Tokushima dataset.

## Discussion

Experienced doctors, when reading medical images, generally follow some patterns and specifically examine a few areas. This study was conducted to improve the phenomenon by which expert doctors’ areas of emphasis and the CNN area of interest differ. Some research efforts have been devised to incorporate a general pattern into CNN as domain knowledge. Nevertheless, these studies were aimed at reaching the state-of-the-art for disease classification. They had not improved it using quantitative equalization. As described herein, we propose an operation branch network leading the network to assign attention to the lung field and heart. Addition of an operation branch reducing the classification accuracy presents difficulties. Therefore, to assess effects on classification accuracy that would be produced by adding the operation branch, we first trained on three chest X-ray datasets: the Teikyo dataset, Tokushima dataset, and NIH14 dataset. Table 2 shows that the Teikyo dataset yielded classification results (93%) and yielded nearly equivalent AUC values (0.98) for ResNet50 and DenseNet121. Furthermore, Table 5 presents NIH 14 dataset results obtained using the proposed method compared to the relevant state-of-the-art method. This proposed method was not better than the state-of-the-art method for the NIH 14 dataset. However, for the Tokushima dataset's pulmonary hypertension (Table 3) and heart failure (Table 4) classification, the operation branch improved AUCs of 0.01 found for the ResNet50 and DenseNet121 networks.

Figure 6 presents examples of attention maps classified as true positive. The attention map of the middle left (original attention branch network) shows anatomical structures such as the lung field, heart, mediastinum, and extracorporeal structures that are unrelated to the diagnosis. These attention maps, particularly addressing the outside of the body, are inappropriate for medical use. However, the attention maps specifically examine the inner regions of weight maps in the operation branch networks (middle right and far-right columns). These results indicate that the operation branch leads the attention map to the appropriate anatomical structures. The feature maps entered in the perception branch are weighted to the attention map, thereby reflecting the anatomical structure.

We calculated the attention index of the Grad-CAM image output by the trained models for quantitative evaluation of the ROI. We created attention index scatter plots to evaluate the degree of improvement by introducing the operation branch. Attention index plots of heart failure, pulmonary hypertension, and the Teikyo dataset are portrayed respectively in Figs. 7, 8, and 9. The upper left dots signify that introducing the operation branch raised the attention index in these figures. Next, as numerical evaluation, we explain the ratio of data with the improved Attention Index. This ratio is the percentage of the number of images for which the Attention Index is improved by our proposed method among the total number of input images. This value corresponds to the number of points located above and to the left of the diagonal of this figure, divided by the total number of points. The proposed methods have achieved 56.5–94.4% for the heart failure classification depicted in Fig. 7 and have achieved 56.7–91.8% for the pulmonary hypertension classification portrayed in Fig. 8. Moreover, the proposed methods have achieved 57.5–83.1% for the Teikyo dataset classification presented in Fig. 9. From these results, we conclude that our proposed method can guide the model in the correct direction for medical use. The operation branch network guided the activated area in the Grad-CAM image successfully to a diagnostically important position. Actually, findings indicate that the ResNet50 results were not as effective as those obtained using DenseNet121.

Figure 10 presents a comparison of Grad-CAM images. From a medical perspective, the activated region is expected to be the area around the heart, but the original attention branch network specifically emphasized areas below the diaphragm and outside the body. By contrast, the operation branch network emphasized the anatomical structures necessary for diagnoses, such as the heart and lung. This figure visually confirms that the operation branch leads the classification network to assign greater attention to the appropriate region than the original attention branch network does.

What is occurring to produce the data shown below the diagonal line in the scatter plot of the attention index (Figs. 7, 8 and 9)? A comparison of the Grad-CAM images is presented in Fig. 11. The activated area in the upper images has moved from the left ventricle (upper center) to the right diaphragm (upper right), whereas the lower image's activated area moved from the superior vena cava (lower center) to the region around the heart (lower right). These figures suggest that decreasing the attention index does not mean that the attention region moves outside of the appropriate position in the chest X-ray image.

This method can also be applied to other modalities. For example, from magnetic resonance images, pneumonia, nodules, and tumors can be detected by particularly addressing the lung field. It is also possible to classify glaucoma in fundus images by particularly emphasizing the optic disk.

An important limitation of the proposed method is that the ROI cannot be guided to a valid region unless the segmentation model's performance is sufficient to create weight maps automatically. As shown in Table 1, the segmentation models in this study have achieved excellent segmentation results when using the Montgomery County X-ray and JSRT datasets, but when applied to the other dataset, because of the influence of domain shift, segmentation accuracy might decrease as a result of the domain shift [56, 57]. This domain shift has the property of increasing in proportion to the distribution difference between the training and test datasets. Manually creating weight maps can prevent this shortcoming, but it is not practical for large-scale data. As an alternative method, one can apply semi-supervised learning, such as Anatomy X-Net [21], to create weight maps simultaneously and automatically with training of the classification models, using a few weight maps as ground truth. Therefore, such semi-supervised learning, which automatically creates weight maps, can solve the domain shift while reducing the cost of creating weight maps.

## Conclusions

This study examined a method of inputting medical knowledge for areas that are observed closely by human physicians when reading chest X-ray images. The method constructs a neural network that assigns attention to useful and important locations for classification. This proposed model requires medical information during training but not during inference. For that reason, it is highly versatile. In addition, this study evaluated the proposed method using a quantitative method to evaluate the degree of improvement in the attention area. The proposed method can maintain or improve classification accuracy, and can enhance capabilities for interpreting images based on later judgment.

## Data Availability

The NIH14 dataset produced during this study is available from the project website at https://nihcc.app.box.com/v/ChestXray-NIHCC. The Teikyo and Tokushima datasets used and analyzed for this study are available from the corresponding author upon reasonable request.

## Abbreviations

CNN: Convolutional neural network
CAM: Class activation mapping
Grad-CAM: Gradient-weighted class activation mapping
AI: Artificial intelligence
ABN: Attention branch network
OBN: Operation branch network
SHAP: Shapley additive explanations
LIME: Local interpretable model-agnostic explanations
